# Detection of torque teno sus virus infection in Indian pigs

**DOI:** 10.14202/vetworld.2019.1467-1471

**Published:** 2019-09

**Authors:** Vinutha Subramanyam, Divakar Hemadri, Shashidhara Phani Kashyap, Jagadish Hiremath, Nagendra Nath Barman, Esther Lalzoliani Ralte, Sharanagouda S. Patil, Kuralayanapalya P. Suresh, Habibur Rahaman

**Affiliations:** 1 ICAR-National Institute of Veterinary Epidemiology and Disease Informatics, Yelahanka, Bengaluru, Karnataka, India; 2 Department of Microbiology and Biotechnology, Jain University, Bengaluru, Karnataka, India; 3 Department of Veterinary Microbiology, College of Veterinary Science, Assam Agriculture University, Guwahati, Assam, India; 4 State Disease Investigation Laboratory, Directorate of Animal Husbandry and Veterinary, Aizawl, Mizoram, India; 5 Regional Representative for South Asia, International Livestock Research Institute, New Delhi, India

**Keywords:** detection, India, phylogeny, polymerase chain reaction, torque teno virus

## Abstract

**Background and Aim:**

Torque teno viruses (TTVs) are circular, single-stranded DNA viruses, which infect a wide range of animals including livestock and companion animals. Swine TTVs (torque teno sus viruses [TTSuVs]) are thought to act as a primary or coinfecting pathogen in pathological conditions such as porcine dermatitis and nephropathy syndrome and post-weaning multisystemic wasting syndrome. So far, the presence of the virus has not been reported in India. Considering that TTSuVs have the potential to cross the species barrier into humans and that pork consumption is common in North-Eastern states of India, the current study aims to investigate the presence of TTSuV in the Indian pig population.

**Materials and Methods:**

A total of 416 samples were collected during 2014-2018, from both apparently healthy pigs and also from pigs suspected of having died from classical swine fever and/or porcine reproductive and respiratory syndrome. These samples were screened for TTSuV infection by polymerase chain reaction (PCR) and DNA sequencing techniques.

**Results:**

The presence of the virus was confirmed in 110 samples from 12 different states of India. Phylogenetic analysis of the nucleotide sequences obtained from the PCR products indicated the presence of viruses of both *Iotatorquevirus* and *Kappatorquevirus* genera in India.

**Conclusion:**

The study is the first report on the presence of TTSuVs in India and highlights the circulation of both genera of the virus in the country.

## Introduction

Torque teno viruses (TTVs) are circular, single-stranded DNA viruses, which infect a wide range of animals including livestock and companion animals [1-3]. Natural infection of pigs with TTV was first described in 1999 [1]. However, a retrospective study from Spain [4] revealed that the virus had been present since at least 1985. Based on nucleotide (NT) sequence homology, TTVs of swine, known as torque teno sus viruses (TTSuVs), are divided into two genera; *Iotatorquevirus* (TTSuV1) and *Kappatorquevirus* (TTSuVk2), with two species in each genus [5].

At present, no clinical signs are specifically associated with TTSuV infection, and no clinicopathological experimental studies involving conventional pigs have been done due to the lack of readily available isolates [6]. However, there is a report that inoculation of gnotobiotic pigs with TTSuV1-containing tissue homogenate caused mild interstitial pneumonia, transient thymic atrophy, membranous glomerulonephropathy, and modest lymphocytic-to-histiocytic liver infiltrates [7]. In addition, there are studies in gnotobiotic pigs, which indicated that swine TTVs, TTSuV1 in particular, can act as a primary or coinfecting pathogen in pathological conditions such as porcine dermatitis and nephropathy syndrome [7,8] and post-weaning multisystemic wasting syndrome [8,9]. Further, there is a report that the rate of detection of TTSuVs in pigs with clinical signs of the coinfecting pathogens is roughly double than that of clinically healthy pigs [10]. In addition, recent studies have reported a high rate of coinfection between TTSuVs and porcine circovirus (PCV)-associated disease including in PCV3 infection [9,11,12]. In India, there are few studies on the presence and distribution of TTVs in the human population [13-15]; nevertheless, the rate of TTV detection has been found to be significantly higher (92.8%) for non-A-E hepatitis group [16]. Until now, there has not been a report on the presence of TTSuVs in animals including pigs in India.

Considering that TTSuVs have the potential to cross the species barrier and infect humans [17] and that pork consumption is common in North-Eastern states of India, this study aims to report the presence of TTSuV in India, based on the screening of tissue specimens/blood/serum/swab samples collected from different parts of the country.

## Materials and Methods

### Ethical approval

This study did not involve the use of live animals, and hence, ethical approval was not required.

### Samples

A total of 416 samples were collected during 2014-2018, from both apparently healthy pigs and also from pigs suspected of having died of classical swine fever and/or porcine reproductive and respiratory syndrome.

### DNA extraction

DNA extraction was performed on individual samples using a commercial DNA extraction kit (DNeasy Blood and Tissue Kit, QIAGEN, Germany) according to the manufacturer’s instructions.

### Polymerase chain reaction (PCR) and DNA sequencing

The PCR was performed using the primers specific for TTSuV1 [18] and TTSuVk2 [19], which targeted a portion of the non-coding region of each viral genome and was expected to produce amplicons of approximately 290 and 230 bases, respectively. Each reaction consisted of 3 µl DNA as template, 1× PCR buffer, 2.5 mM MgCl_2_, 1.0 mM dNTP, 10 pmol forward and reverse primer, 0.25 U Taq DNA polymerase (New England BioLabs, USA), and nuclease-free water. The amplification was performed with the following reaction conditions: A 10 min initial denaturation step at 95°C, followed by 35 cycles at 95°C for 30 s, 55°C for 30 s, and 72°C for 30 s, finishing with 1 cycle for 10 min at 72°C. The PCR products were visualized on a 1.5% agarose gel. The PCR-positive products were purified using the QIAquick PCR Purification Kit (Qiagen, Hilden, Germany) according to the manufacturer’s instructions and eluted in 30 μl of elution buffer. The representative amplified products were cloned into the pGEM-T vector (Promega, USA) and subjected to NT sequencing using M13 primers (Eurofins Scientific, India).

### Phylogenetic analysis

Phylogenetic analysis was carried out using MEGA X software [20]. Briefly, the NT sequences of TTSuVs generated in the present study were aligned and analyzed with the prototype sequences of all of the four presently defined species of the virus and several additional sequences available in the NCBI GenBank database.

## Results

### PCR

TTSuV was detected in pigs from 12 of the 16 different states investigated and a total of 110 samples were positive of the 416 samples screened. Of those samples which were positive, 40 were positive for TTSuV1, while 70 were positive for TTSuVk2. The details of the samples are given in Table-1.

**Table 1:** Details of samples screened for TTSuV infection in domestic pigs.

S. No.	State	Number of screened	Type of material				Number of TTSuV1 positive	Number of TTSuV2 positive

			Tissue	Blood	Nasal swab	Serum		
1	Andhra Pradesh	6	0	6	0	0	0	0
2	Arunachal Pradesh	21	0	0	0	21	0	0
3	Assam	20	14	6	0	0	1	1
4	Chhattisgarh	10	0	0	0	10	2	2
5	Goa	38	13	4	13	8	4	6
6	Karnataka	105	28	66	0	11	14	23
7	Kerala	26	2	0	0	24	1	1
8	Madhya Pradesh	27	6	2	0	19	1	2
9	Maharashtra	37	11	2	3	21	4	14
10	Manipur	14	0	0	0	14	0	0
11	Meghalaya	4	1	0	0	3	1	1
12	Mizoram	33	13	13	0	7	3	3
13	Odisha	7	7	0	0	0	0	2
14	Punjab	13	0	3	7	3	0	0
15	Sikkim	42	0	42	0	0	6	7
16	Telangana	13	7	6	0	0	3	8
Total		416	102	150	23	141	40	70

TTSuV=Torque Teno Sus Virus

### Phylogenetic analysis

**Figure-1:**
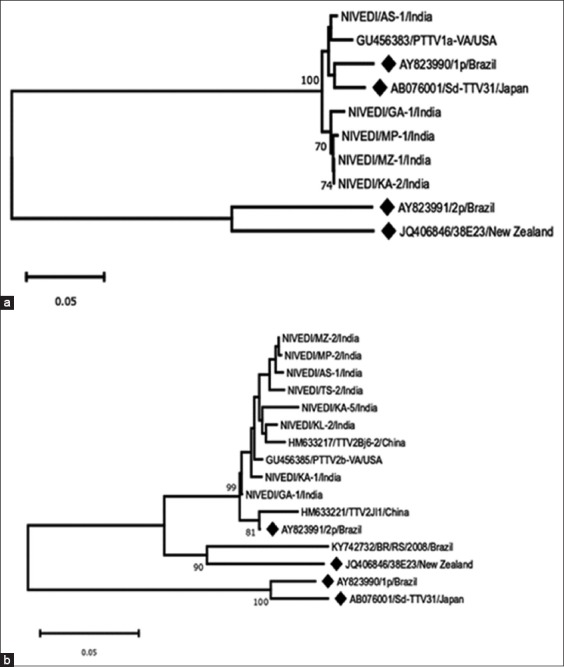
Neighbor-joining tree showing the grouping of Indian torque teno sus viruses (TTSuVs) with the prototype species (♦) of the genus, *Iotatorquevirus* (a): TTSuV1a (AB076001) and TTSuV1b (AY823990) and *Kappatorquevirus* (b): TTSuV K2a (AY823991) and TTSuV K2b (JQ406846). The percentage of replicate trees in which the associated taxa clustered together in the bootstrap test (1000 replicates) is shown next to the branches. The tree was drawn to scale, with branch lengths in the same units as those of the evolutionary distances used to infer the phylogenetic tree. The evolutionary distances were computed using the p-distance method and are in the units of the number of base differences per site.

Phylogenetic analysis of the NT sequences obtained from the above PCR products of TTSuV is shown in Figure-1a and b. From Figure-1b, it can be seen that all the NT sequenced TTSuVs of the *Kappatorquevirus* genus in our study belong to the species, TTSuVk2a; on the contrary, the species identity of the TTSuVs of the *Iotatorquevirus* genus circulating in India could not be determined as their grouping on the phylogenetic tree (Figure-1a) was not sufficiently resolved.

## Discussion

In all, of the 416 samples screened for TTSuV, 110 samples were positive, of which 40 were positive for TTSuV1, while 70 were positive for TTSuVk2. Earlier, we made attempts to amplify TTSuV1 using the primer pair reported by Kekarainen [19]. However, these primers failed to detect TTSuV1 in our samples. In contrast, TTSuV1 was successfully detected in our samples using the primer pair reported by Ozawa *et al*. [18]. A comparison of the primer sequences revealed that the reverse primer used by Ozawa *et al*. [18] though almost identical to that of Kekarainen [19] differs by three NT bases at the 3’ end. The difference in the NT composition of Indian TTSuV1 in these positions could be the reason for the failure in our earlier attempts. NT sequencing of the representative PCR-amplified products further confirmed that these were, in fact, specific for TTSuVs. Among the samples screened from 16 Indian states (Table-1), it could be seen that samples from 10 states were positive for both the TTSuV genera. Samples from the states of Odisha and Kerala were negative for TTSuV1, while those from Andhra Pradesh, Arunachal Pradesh, Sikkim, and Punjab were negative in both the genus-specific PCRs. Considering the variable prevalence (17-100%) of TTSuVs globally [6], screening of a larger number of samples from the above states would be required to accurately assess their prevalence in these regions. It is worth noting that although TTSuVs have been detected in different organs, relatively high detection rates have been found in the kidneys of apparently healthy slaughterhouse aged pigs [21]. Among the various types of samples screened, 35.3, 28.7, 16.3, and 34.8% of tissue specimens, blood, serum, and nasal swabs, respectively, were positive for the presence of TTSuVs. Phylogenetic analysis of the NT sequences obtained from the above PCR products of TTSuV is shown in Figure-1a and b. From Figure-1b, it can be seen that all of the NT sequenced TTSuVs of the *Kappatorquevirus* genus in our study belong to the species, TTSuVk2a; on the contrary, the species identity of TTSuVs of the *Iotatorquevirus* genus circulating in India could not be determined as their grouping on the phylogenetic tree (Figure-1a) was not resolved sufficiently. It has been reported that non-coding regions contain NT sequences which are highly conserved among TTVs infecting humans, non-human primates, and other animals [2], as this region harbors regulatory sequences important in replication and transcription [22], and promoter and enhancer elements that exhibit different transcriptional activities in different cell lines [23,24]. Here, we used primers located in non-coding regions, wherein sequence homologies of 91-97% and 93-99% were noted for the TTSuV1 and TTSuVk2 genogroups, respectively [18]. Therefore, it is quite plausible that the NT divergence observed in the PCR-amplified region (260-290 bases) was not sufficient for species classification of *Iotatorquevirus* by phylogenic techniques.

## Conclusion

In the present study, we have screened specimens from diverse and distant geographical regions of India and the detection of TTSuVs in these specimens indicated the widespread presence of these viruses across the country. Further studies involving more genomic sequences of the coding region are required to provide a more accurate assessment of the prevalence of TTSuVs circulating in India. Nevertheless, the present study has provided us with first-hand knowledge of the circulation of TTSuVs in India and also that the viruses of both genera are prevalent.

## Authors’ Contributions

VS, DH, KPS, and HR conceived and designed the experiments. DH wrote the manuscript, while JH and SSP assisted in writing the manuscript. VS, SPK, NNB, and ELR performed the experiments. All authors read and approved the final manuscript.
